# *In vitro* and *in vivo* characterization of a bat merbecovirus with ACE2- and DPP4-independent cell entry

**DOI:** 10.1128/jvi.00727-25

**Published:** 2025-06-17

**Authors:** Hiromichi Matsugo, Tomoya Kitamura, Naohiro Takahashi, James Chambers, Ayano Ichikawa, Misa Katayama, Kaixin Li, Wataru Sekine, Kosuke Ohira, Hiroho Ishida, Akiko Takenaka-Uema, Kazuyuki Uchida, Masayuki Shimojima, Taisuke Horimoto, Shin Murakami

**Affiliations:** 1 Laboratory of Veterinary Microbiology, Graduate School of Agricultural and Life Sciences, University of Tokyo 13143, Tokyo, Japan; 2 African Swine Fever Unit, National Agriculture and Food Research Organization (NARO), National Institute of Animal Health 34777, Tokyo, Japan; 3 Laboratory of Veterinary Pathology, Graduate School of Agricultural and Life Sciences, University of Tokyo 13143, Tokyo, Japan; 4 Department of Virology I, National Institute of Infectious Diseases 13511, Tokyo, Japan; University of North Carolina at Chapel Hill, Chapel Hill, North Carolina, USA

**Keywords:** receptor, reverse genetics, bat, merbecovirus, coronavirus

## Abstract

Betacoronaviruses, which have caused three human outbreaks within the last two decades, are thought to originate from bats, raising the concern that bat coronaviruses could cause a novel human outbreak in the future. To determine whether the bat merbecovirus EjCoV-3 strain, previously detected in *Eptesicus japonensis* in Japan, has the potential to infect humans, we analyzed its cellular entry mechanism. Cellular entry of EjCoV-3 via the spike protein requires protease treatment and is mediated by an unknown receptor, other than DPP4 or ACE2. We generated cultivable recombinant EjCoV-3 using bacterial artificial chromosome-based reverse genetics and found that it efficiently replicated in human respiratory and intestinal cell cultures as well as nasal ciliated epithelium in hamsters. These findings suggest that bat merbecovirus with ACE2- and DPP4-independent cell entry has the potential to cause human infections, highlighting the importance of extensive bat surveillance for pandemic preparedness.

**IMPORTANCE:**

Betacoronaviruses, including severe acute respiratory syndrome coronavirus (SARS-CoV), Middle East respiratory syndrome coronavirus (MERS-CoV), and SARS-CoV-2, have caused three significant outbreaks in the past two decades and are believed to have originated from bats. To investigate the potential for future outbreaks, we generated a Japanese bat-derived MERS-related coronavirus, designated EjCoV-3, using reverse genetics. Our results showed that EjCoV-3 does not utilize ACE2 and DPP4, cell entry receptors for SARS-CoV and MERS-CoV, as a means of infection. However, we found that EjCoV-3 is the first bat merbecovirus capable of efficiently replicating in human respiratory cells and the respiratory tract of hamsters. These findings provide new insight into the potential for MERS-related coronaviruses that do not use ACE2 and DPP4 to infect the human respiratory tract, highlighting the importance of preparedness for outbreaks caused by these viruses.

## INTRODUCTION

Coronaviruses are classified into four genera: *Alphacoronavirus*, *Betacoronavirus*, *Gammacoronavirus*, and *Deltacoronavirus*. Among these, the betacoronaviruses include severe acute respiratory syndrome coronavirus (SARS-CoV), SARS-CoV-2, and Middle East respiratory syndrome coronavirus (MERS-CoV). SARS-CoV-2 caused coronavirus disease 2019, which devastated global health and the economy. Human outbreaks of these highly pathogenic betacoronaviruses have occurred in a short period over the last 20 years, raising concern about the occurrence of a novel betacoronavirus outbreak in the near future. These emerging betacoronaviruses have been considered to originate from bats (1, 2), requiring an assessment of the spillover potential of bat betacoronaviruses to humans.

MERS-CoV, belonging to the subgenus *Merbecovirus*, has infected 2,613 and killed 943 individuals (36% mortality rate) since it was first identified in Saudi Arabia in 2012 (3). Because dromedary camels (*Camelus dromedarius*) in Saudi Arabia harbor viruses that are approximately genetically identical to MERS-CoV, the camels were considered the source of human infection (4). Further epidemiological studies showed high seroprevalence in dromedary camels in the Arabian Peninsula (5–8), suggesting that camels are natural reservoirs of MERS-CoV. Merbecoviruses have also been detected in multiple bat species belonging to the families *Vespertilionidae*, pangolins, and hedgehogs (9–16). Among bat merbecoviruses, the NeoCoV strain, which was detected in *Neoromicia capensis* in South Africa, was most closely related to MERS-CoV (9). However, the sequence homology of the spike (S) gene between the two viruses was lower than that of other genomic regions, suggesting that the NeoCoV strain cannot be the direct ancestor of MERS-CoV and that S gene recombination with unknown viruses may be required for zoonosis.

MERS-CoV initiates infection in humans by binding the S protein to the cellular receptor, dipeptidyl peptidase-4 (DPP4). Certain bat and pangolin merbecoviruses, such as HKU4 (17–19), HKU25 (13), and NL140422 (15), also use human DPP4 as a receptor in cell culture. However, the HKU4 (SM3A strain) reportedly utilizes an unknown bat-specific receptor (20). In contrast, African merbecoviruses, NeoCoV and PDF-2180, utilize bat ACE2 as a receptor (21). Moreover, recent studies have demonstrated that additional merbecoviruses can utilize either bat or human ACE2 as a receptor (22–24). These findings suggest that the receptor usage of bat merbecoviruses is diverse and complex. After binding of the betacoronavirus to cell receptors, cleavage of the S protein by cellular proteases is required for membrane fusion to release the virus genomes into the cytoplasm. The S protein of MERS-CoV is cleaved by furin or related proprotein convertases within virus-producing cells, and during cell entry, it is cleaved by the host serine protease TMPRSS2 at the plasma membrane or by cathepsin B/L in the endosomes (25–28). Pseudotyped virus-based assays revealed that bat merbecoviruses HKU4 (17, 18), HKU25 (13), NeoCoV, and PDF-2180 (21) were activated by exogenous trypsin in cell culture. However, the HKU4 SM3A strain does not require exogenous trypsin to infect human cells (20). Therefore, the involvement of cellular proteases, such as TMPRSS2, and the mechanism for cleavage activation of the S proteins of bat merbecoviruses remains to be elucidated in detail.

Although pseudotyped viruses are efficient tools for studying the entry mechanism of viruses that have not been isolated, it is difficult to analyze the characteristics of viral replication *in vitro* and *in vivo*. Moreover, in the case of sarbecoviruses, infectivities occasionally differed between replication-competent and pseudotyped viruses (29), suggesting the necessity of authentic cultivable viruses for entry studies. However, there are few reports on the isolation of cultivable bat merbecoviruses, and their potential to infect other mammals, including humans, has remained unevaluated (20, 23, 30). We previously detected a bat merbecovirus, EjCoV-3 strain, in *Eptesicus japonensis* in Japan and characterized its genetic characteristics (16). However, virological properties, such as cellular and organ tropism, which are important for assessing its spillover potential, remain unknown.

Here, we determined the protease requirement for the entry of EjCoV-3 using a pseudotyped virus with its S protein. Based on these findings, we established a reverse genetics system with effective culture methods for EjCoV-3 and analyzed the growth characteristics of the rescued virus in several human cell cultures and animal models.

## RESULTS

### Effect of exogenous proteases on cell entry of the pseudotyped virus containing the EjCoV-3 S protein

**Fig 1:**
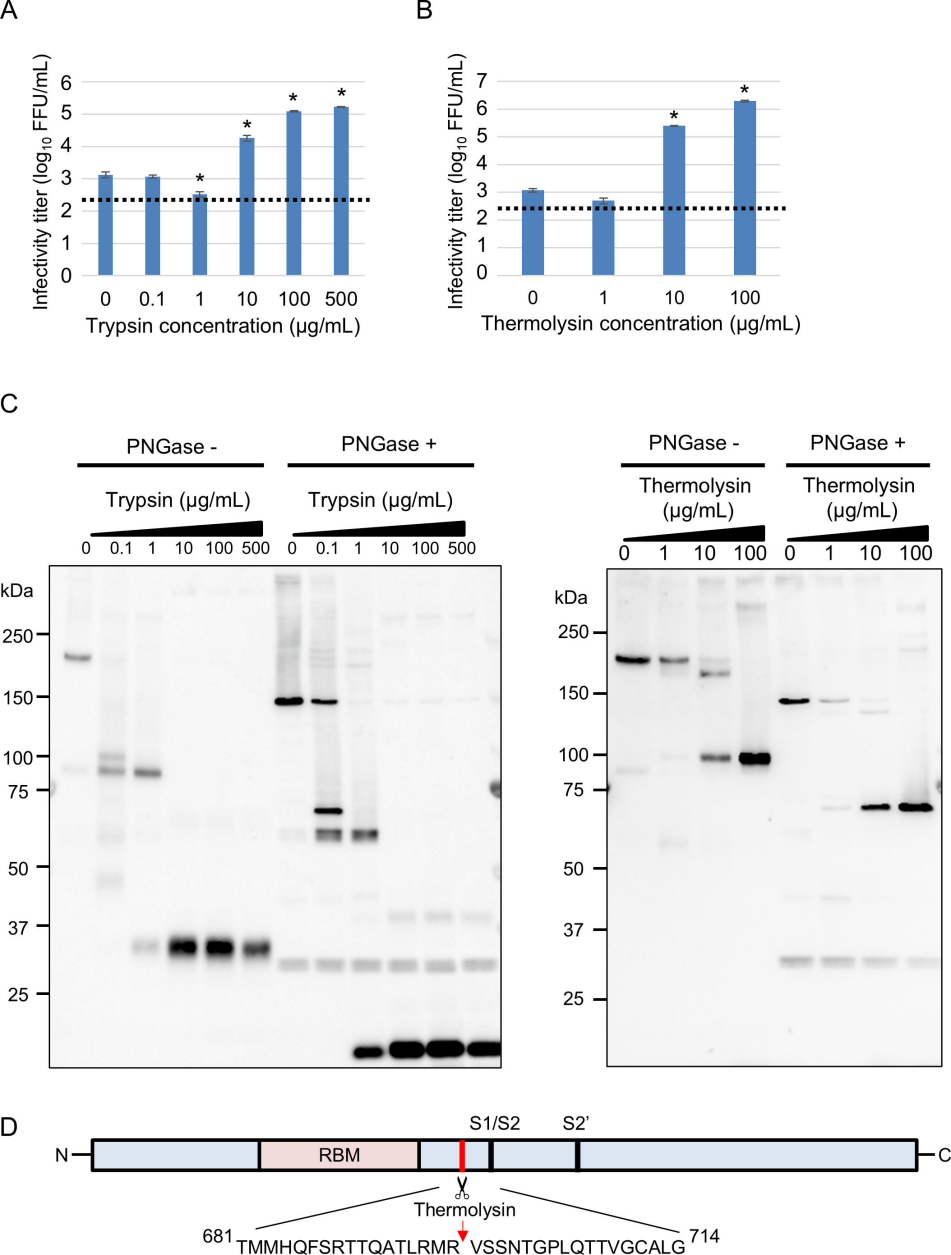
Characterization of protease-treated VSV/EjCoV-3. Vero/TMPRSS2 cells were inoculated with VSV/EjCoV-3 treated with various concentrations of trypsin (**A**) or thermolysin (**B**). At 20 h post-infection, the infectivity titers were calculated by counting GFP-positive cells. The results are reported as the mean titers with standard errors for three independent experiments. The asterisks indicate significant differences compared to untreated VSV/EjCoV-3 (*P < 0.01 by Dunnett’s test). A dotted line indicates the detection limit. (**C**) Proteolysis of VSV/EjCoV-3 S protein was examined following treatment with various concentrations of trypsin or thermolysin with (+) or without (−) peptide-N-glycosidase F (PNGase). The C-terminal fragments of FLAG-tagged S proteins were detected by anti-FLAG antibody by western blotting analysis. (**D**) The thermolysin-mediated cleavage site of the EjCoV-3 S protein. The FLAG-tagged S proteins were isolated from thermolysin-treated VSV/EjCoV-3. N-terminal amino acid sequencing was performed using a protein sequencer.

Upon entry into cells, coronaviruses require their S proteins to be cleaved by cellular or exogenous proteases. Previous studies have reported that the host protease TMPRSS2 cleaves and activates the S proteins of betacoronaviruses, such as SARS-CoV (31), SARS-CoV-2 (32), and MERS-CoV (26, 27). To test whether exogenous proteases can enhance the entry of EjCoV-3 into Vero/TMPRSS2 cells, we generated vesicular stomatitis virus (VSV) pseudotyped with a C-terminal FLAG-tagged S protein of EjCoV-3 (VSV/EjCoV-3) and analyzed the requirement of trypsin or thermolysin for cell entry (Fig. 1A and B). We observed that viral infectivity significantly increased in the presence of high concentrations of trypsin (10, 100, and 500 µg/mL) and thermolysin (10 and 100 µg/mL), although their low concentrations (0.1 or 1 µg/mL) did not. These data suggested that proteolytic cleavage of the S protein is required for VSV/EjCoV-3 to enter cells efficiently.

To examine the cleavage profiles of protease-treated S protein of EjCoV-3, we treated VSV/EjCoV-3 with trypsin or thermolysin, followed by incubation with the glycosidase PNGaseF, and detected C-terminal fragments of the cleaved S proteins using western blotting with an anti-FLAG antibody (Fig. 1C). Treatment with lower concentrations of trypsin (0.1 and 1 µg/mL) generated 102 and/or 88 kDa fragments (68 and/or 58 kDa fragments in deglycosylated forms), whereas treatment with higher concentrations of trypsin (10, 100, and 500 µg/mL) generated 32 kDa and minor 61 kDa fragments (17 kDa and 43 kDa in the deglycosylated forms). In contrast, treatment with an increasing concentration of thermolysin generated a 94 kDa fragment (65 kDa in deglycosylated form). Since a clear correlation was observed between prominent cleavage by thermolysin and enhanced infectivity, we further investigated the cleavage site using N-terminal sequencing. The analysis revealed that thermolysin cleaves at a site located between the putative receptor-binding domain and the S1/S2 cleavage site (Fig. 1D). These data indicate that cleavage profiles differ between the two proteases, suggesting distinct protease-specific activation mechanisms during EjCoV-3 cell entry.

### Requirement of host proteases for VSV/EjCoV-3 infection

**Fig 2:**
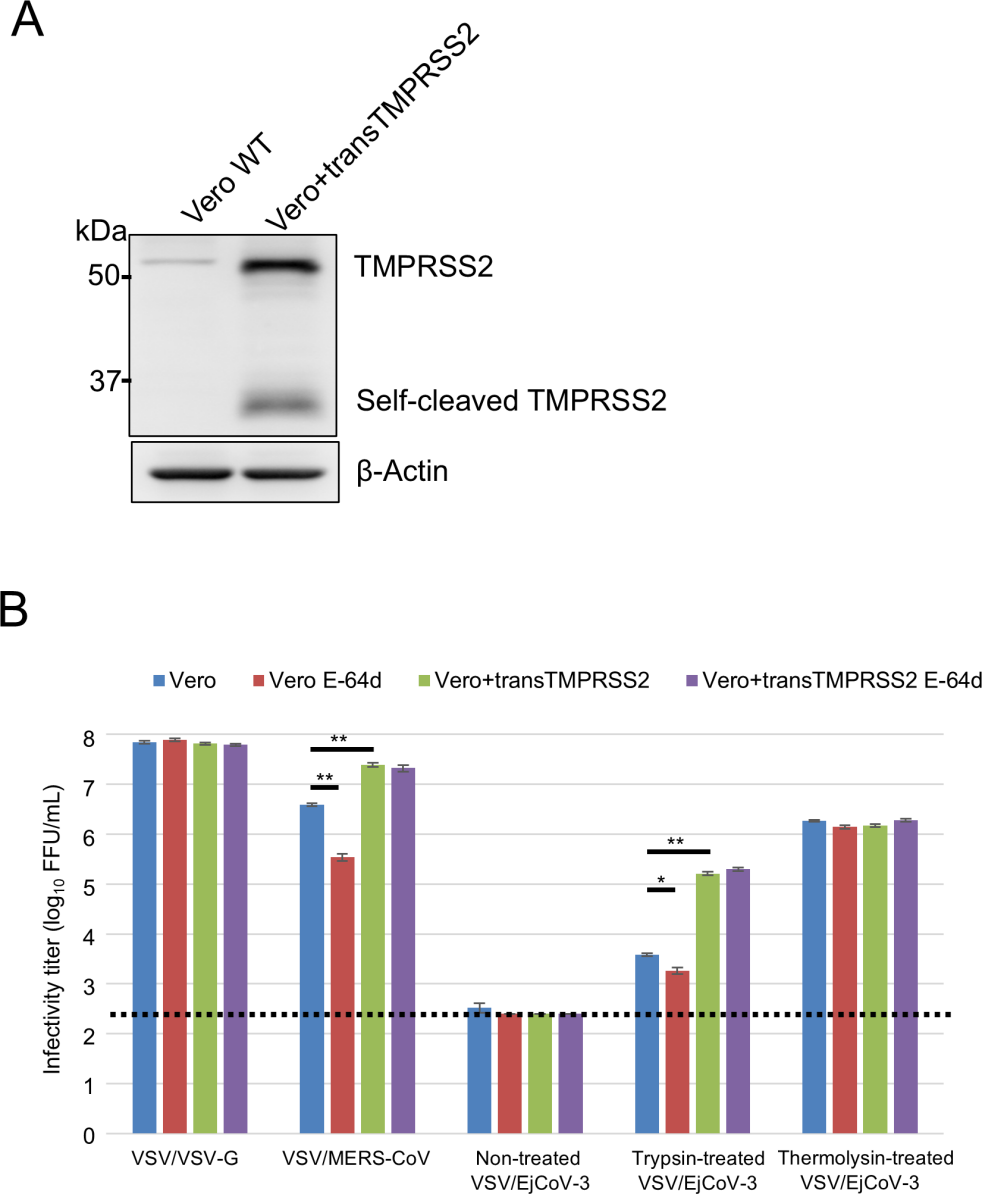
Requirement of TMPRSS2 and cathepsins for cell entry of trypsin-treated VSV/EjCoV-3. (**A**) TMPRSS2 expression in Vero+transTMPRSS2 cells by lentivirus vector was confirmed by western blotting analysis using anti-TMPRSS2 antibody. (**B**) The effects of cathepsins B/L and TMPRSS2 on VSV/EjCoV-3 infection were examined in Vero cells and Vero+transTMPRSS2 cells using cathepsin inhibitor E-64d. After incubation of E-64d (25 µM) for 2 h at 37°C, the cells were infected with untreated, trypsin-treated (500 µg/mL), and thermolysin-treated (100 µg/mL) VSV/EjCoV-3, or VSV/VSV-G and VSV/MERS-CoV as controls. At 20 h post-infection, the infectivity titers were calculated by counting GFP-positive cells. The results are reported as the mean titers with standard errors for three independent experiments. The asterisks indicate significant differences (*P < 0.05; **P < 0.01 by the Holm method). A dotted line indicates the detection limit.

A previous study indicated that one of the bat merbecoviruses, HKU4, is not activated by human TMPRSS2 (hTMPRSS2) (17). To test whether exogenous protease-treated EjCoV-3 requires hTMPRSS2, to directly assess the role of hTMPRSS2 in viral entry, we prepared Vero cells transduced with the hTMPRSS2 gene through a lentiviral vector (Vero+transTMPRSS2 cells). A high level of hTMPRSS2 containing the self-cleaved form was expressed in Vero+transTMPRSS2 cells compared with the parental wild-type Vero cells (Fig. 2A). When we infected wild-type Vero or Vero+transTMPRSS2 cells with trypsin-treated VSV/EjCoV-3, significantly high infectivity was found in the presence of hTMPRSS2, similar to the control VSV/MERS-CoV, which possessed MERS-CoV S (Fig. 2B). This suggested that both trypsin and hTMPRSS2 are synergistically responsible for the efficient entry of VSV/EjCoV-3 into cells. In contrast, the infectivity of thermolysin-treated VSV/EjCoV-3 did not increase in the presence of hTMPRSS2, as did the control VSV/VSV-G strain, which possessed VSV-G (Fig. 2B).

Previous reports have shown that MERS-CoV S is activated by human cathepsin B and L (26), in addition to hTMPRSS2, whereas HKU4 S is not (17, 33). Thus, using the cathepsin inhibitor E-64d, which reportedly blocks MERS-CoV entry into Vero cells, we assessed the requirement of cathepsins for the entry of VSV/EjCoV-3 (34, 35). E-64d treatment significantly blocked the entry of trypsin-treated VSV/EjCoV-3 into Vero cells, similar to VSV/MERS-CoV, but not like the thermolysin-treated cells, such as VSV/VSV-G (Fig. 2B). These data suggested that the trypsin-treated S protein of EjCoV-3 was partially activated by cathepsins in Vero cells.

### Activation of the EjCoV-3 S protein after viral attachment by trypsin treatment

**Fig 3:**
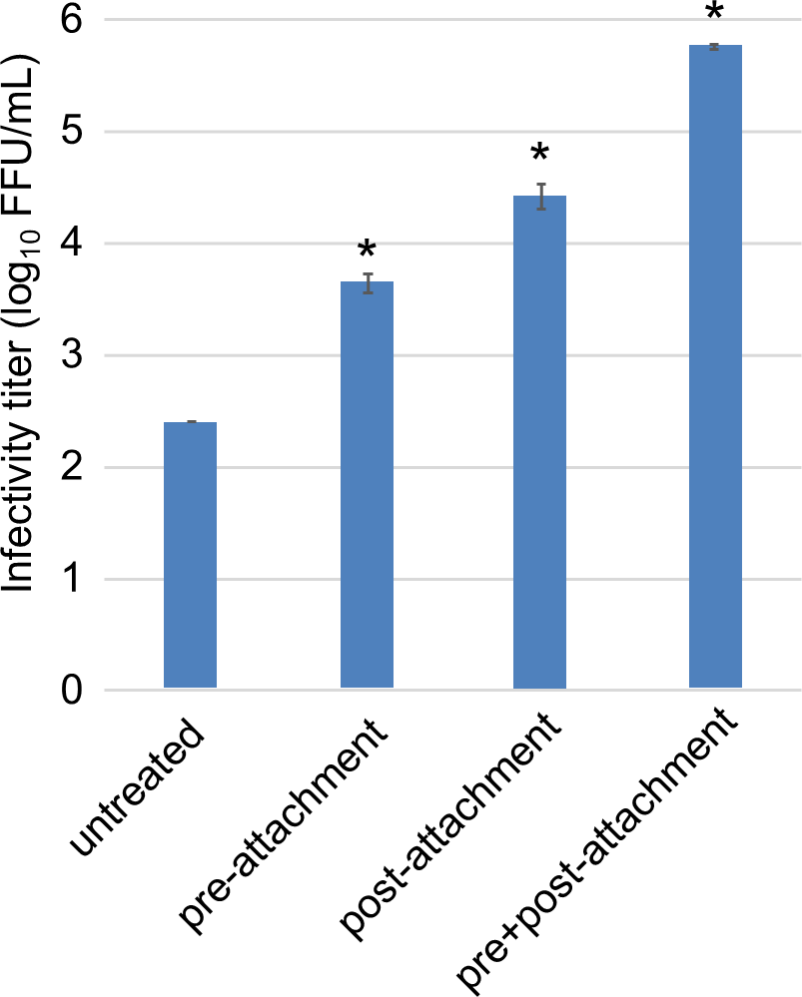
Enhancement in infectivity of VSV/EjCoV-3 by trypsin treatment for pre- or post-viral attachment on the cell surface. After being pre-incubated for 15 min on ice, Vero cells were inoculated and incubated with trypsin-treated (500 µg/mL) (for pre- or pre+post-attachment virus) or untreated VSV/EjCoV-3 (for untreated or post-attachment virus) for 1 h on ice. Then, the cells were incubated at 37°C in the presence (for post- or pre+post-attachment virus) or absence (for untreated or pre-attachment virus) of trypsin. At 20 h post-infection, the infectivity titers were determined by counting GFP-positive cells. The results are reported as the mean titers with standard errors for three independent experiments. Asterisks indicate significant differences compared to the control (*P < 0.05 by the Holm method).

The S proteins of various coronaviruses undergo conformational changes by binding to their receptors, facilitating access to proteases (28, 35–37). We examined the infectivity of VSV/EjCoV-3 through trypsin treatment before and after attachment to Vero cells. Post-attachment treatment with trypsin resulted in a significantly higher infectivity titer of VSV/EjCoV-3 (Fig. 3). The combination of pre- and post-attachment treatments further enhanced the infectivity of VSV/EjCoV-3. These data suggested that, similar to other coronaviruses, binding of the S protein of EjCoV-3 to the cell surface enhances protease access.

### DPP4- and ACE2-independent entry of the VSV/EjCoV-3

**Fig 4:**
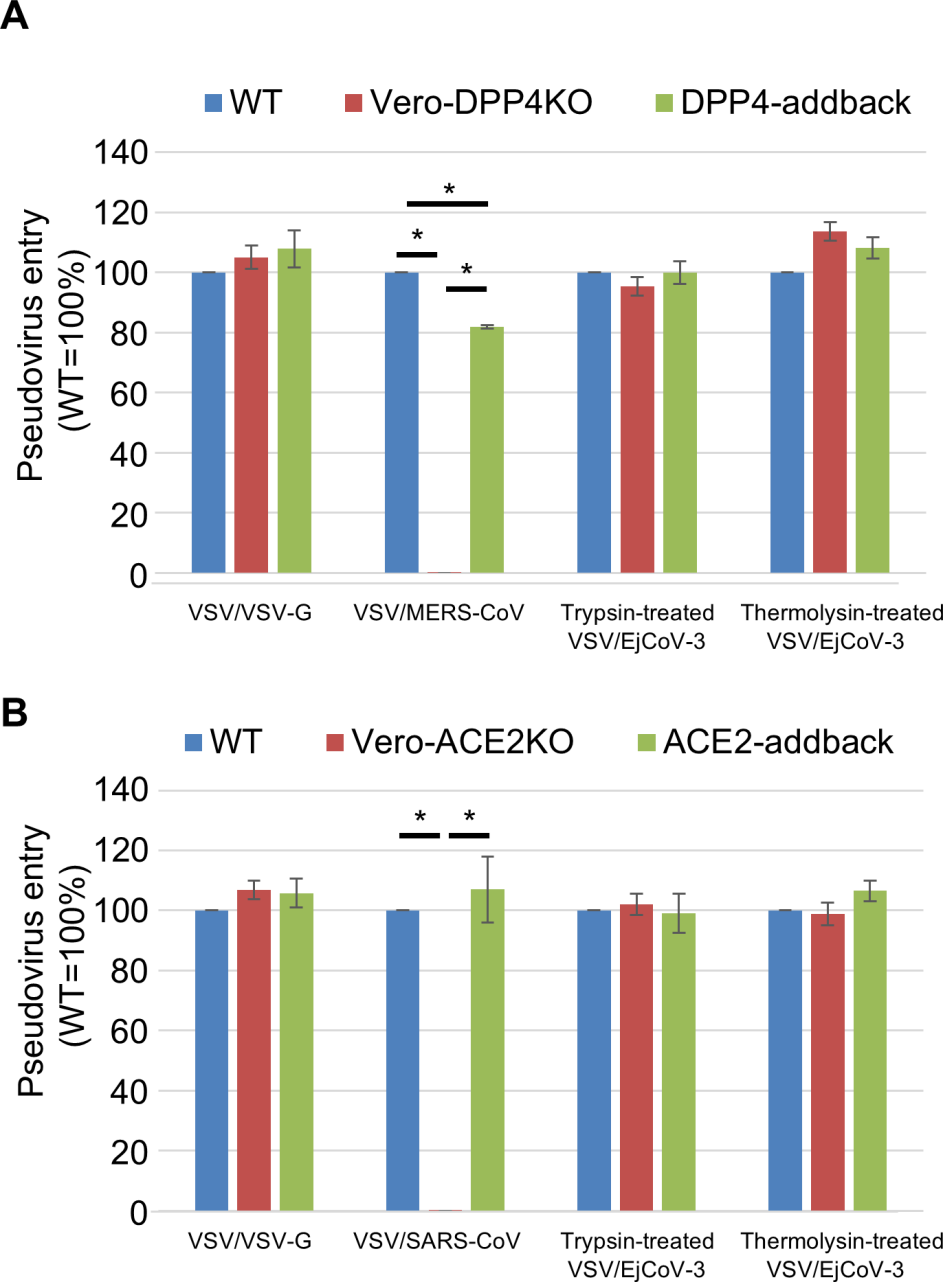
Cell entry of VSV/EjCoV-3 to DPP4- or ACE2-knockout cells. (**A**) Vero/TMPRSS2 (WT), Vero-DPP4KO, and DPP4-addback cells were inoculated with trypsin-treated and thermolysin-treated VSV/EjCoV-3, or VSV/VSV-G and VSV/MERS-CoV as controls. (**B**) WT, Vero-ACE2KO, and ACE2-addback cells were inoculated with trypsin-treated and thermolysin-treated VSV/EjCoV-3, or VSV/VSV-G and VSV/SARS-CoV as controls. At 20 h post-infection, the infectivity titers were calculated by counting GFP-positive cells. The infectivity titers were normalized to that of WT. The results are reported as the mean titers with standard errors for three independent experiments. The asterisks indicate significant differences (*P < 0.01 by the Holm method).

To examine whether EjCoV-3 uses DPP4 or ACE2 as an entry receptor, we established endogenous DPP4 or ACE2 knockout Vero/TMPRSS2 cells (38) using the CRISPR/Cas system (Vero-DPP4 or Vero-ACE2KO cells, respectively). Using lentivirus vectors, we also prepared addback cells with transduced DPP4 or ACE2 genes (referred to as DPP4-addback or ACE2-addback cells, respectively). Both the knockout of DPP4 or ACE2 and the addback were confirmed through the western blot analysis (Fig. S1). We then inoculated these cells with trypsin- or thermolysin-treated VSV/EjCoV-3 and counted the number of GFP-positive cells after 20 h of incubation (Fig. 4A and B). The infectivities of VSV/MERS-CoV and VSV/SARS-CoV, both used as controls, were below the detection limit for measurement in Vero-DPP4KO and Vero-ACE2KO cells, respectively, and were restored in the corresponding addback cells. In contrast, no significant reduction in the infectivity of trypsin- or thermolysin-treated VSV/EjCoV-3 cells was observed, suggesting that EjCoV-3 uses an undefined receptor molecule other than ACE2 and DPP4.

### Rescue of an infectious EjCoV-3 from the BAC clone

**Fig 5:**
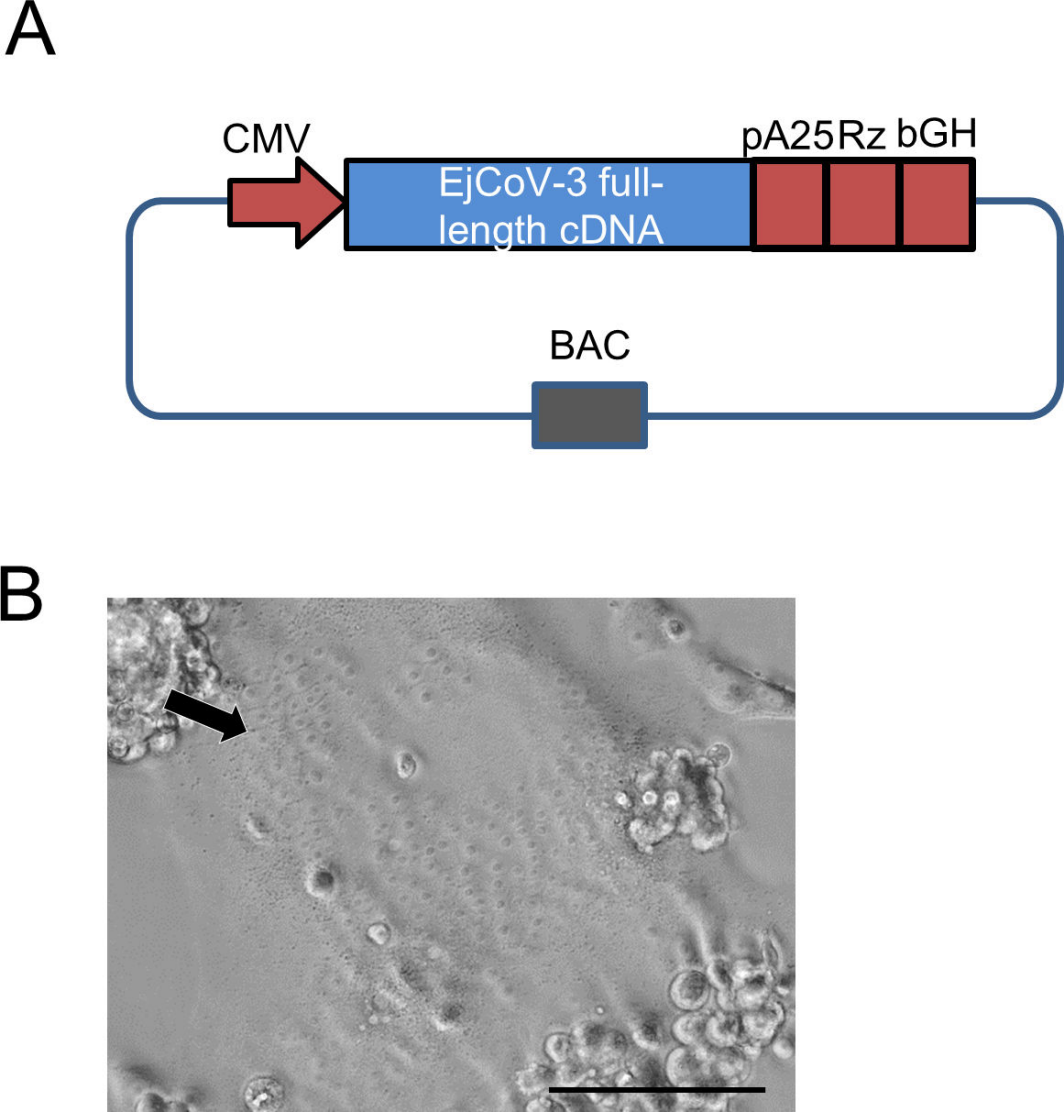
Rescue of EjCoV-3 from a BAC clone. (**A**) Schematic representation of the infectious BAC clone was shown. Cytomegalovirus immediate early promoter (CMVp), the full-length cDNA of EjCoV-3, a 25-nucleotide poly A (pA), hepatitis delta virus ribozyme (Rz), and bovine growth hormone polyadenylation signal (bGH) were cloned into BAC. (**B**) Photo image of EjCoV-3-infected cells was shown. Vero/TMPRSS2 cells were infected with the rescued EjCoV-3 and incubated in the medium with 10 µg/mL trypsin. Clear large syncytia formation (arrow) was observed on 2 days post-infection. The scale bar indicates 100 µm.

Since we were unable to isolate the authentic virus through cell culture, including Vero/TMPRSS2 cells, we employed reverse genetics to generate infectious EjCoV-3. To establish a reverse genetics system for EjCoV-3, we cloned the entire EjCoV-3 genome into a bacterial artificial chromosome (BAC) clone (pBAC-EjCoV-3) (Fig. 5A; Fig. S2). HEK293T cells were transfected with pBAC-EjCoV-3, and the supernatant was collected 2 days post-transfection. The thermolysin (100 µg/mL)-treated supernatant was inoculated into Vero/TMPRSS2 cells. At 12 h post-inoculation, the medium was replaced with trypsin (10 µg/mL)-supplemented medium. Clear cytopathic effects containing large syncytia were observed 2 days post-infection (Fig. 5B). The supernatant was passed thrice to amplify the virus, reaching a viral titer of 5.4 × 10^5^ PFU/mL. Sanger sequencing of the full-length EjCoV-3 genome confirmed the absence of mutations. These results indicate that an unmutated, infectious EjCoV-3 was successfully rescued using reverse genetics.

### Requirement of proteases for efficient replication of EjCoV-3 in cell culture

**Fig 6:**
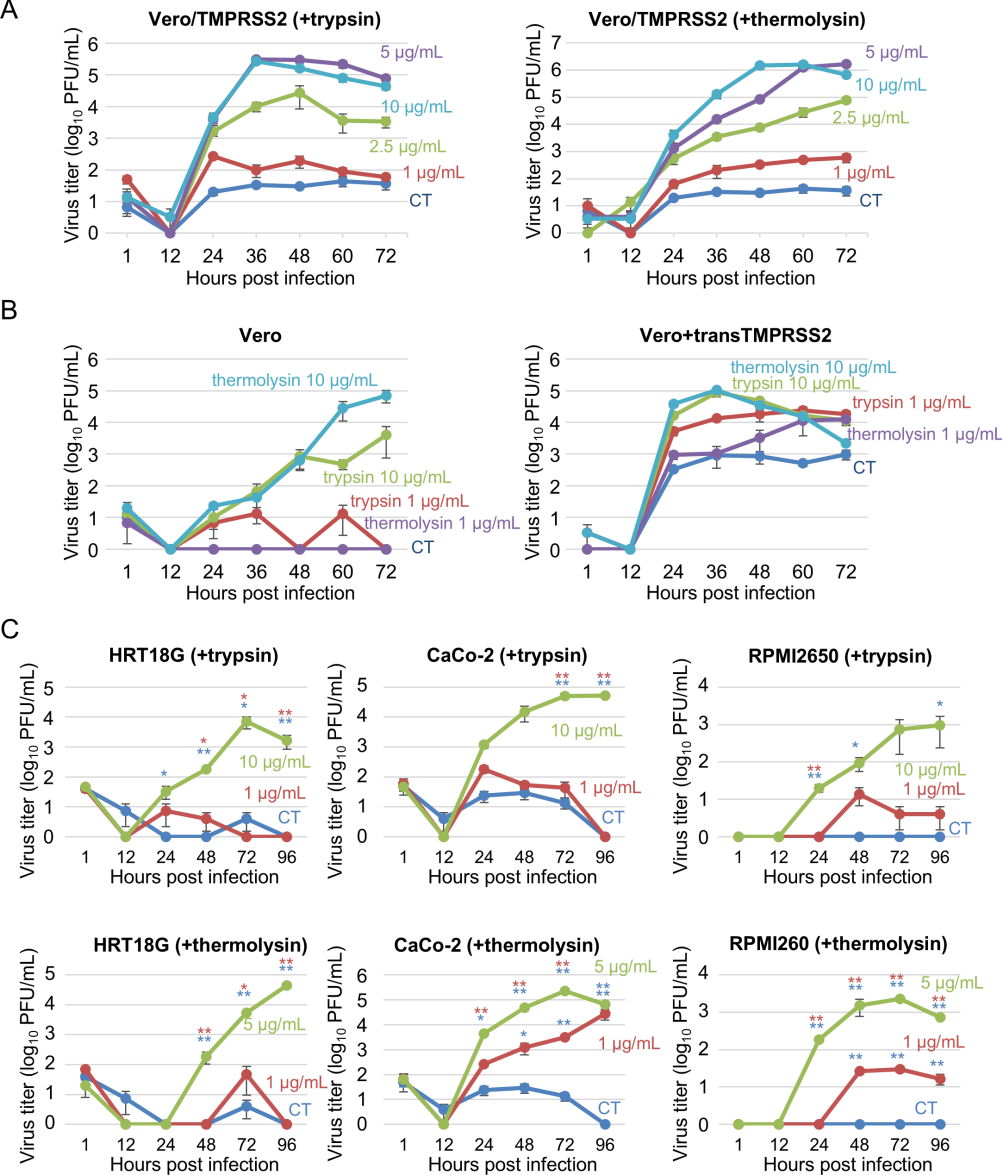
Growth kinetics of EjCoV-3 in cell cultures. (**A**) Vero/TMPRSS2 and (**B**) Vero or Vero+transTMPRSS2 cells were infected with EjCoV-3 at a multiplicity of infection (MOI) of 0.01. (**C**) HRT18G, CaCo-2, or RPMI2650 cells were infected with EjCoV-3 at an MOI of 0.1. The infected cells were cultured, and at 12 h post-infection, the medium was exchanged for one with or without various concentrations of trypsin or thermolysin. The culture supernatants were collected at the indicated time points, and the viral titers were determined using the plaque assay. The results are reported as the mean titers with standard errors for three independent experiments. Blue and red asterisks indicate significant differences compared to control (CT) and that with 1 µg/mL trypsin or thermolysin, respectively (*P < 0.05; **P < 0.01 by the Holm method).

We tested protease dependency for the infectivity of rescued EjCoV-3, as observed in pseudotype virus-based experiments. We first examined the growth kinetics of EjCoV-3 in Vero/TMPRSS2 cells treated with various concentrations of trypsin or thermolysin (Fig. 6A). EjCoV-3 efficiently replicated in a dose-dependent manner with varying concentrations of the proteases. Next, to directly verify the requirement of TMPRSS2 in EjCoV-3 infectivity, we compared its growth in Vero with that in Vero+transTMPRSS2 cells expressing hTMPRSS2 in *trans* using a lentiviral vector system, with or without exogenous proteases (Fig. 6B). In the presence of no or low concentrations (1 µg/mL) of trypsin or thermolysin, EjCoV-3 exhibited low replication in Vero cells, whereas it replicated at a considerably higher rate under high concentrations (10 µg/mL) of proteases. In contrast, EjCoV-3 replicated more efficiently in Vero+transTMPRSS2 cells than in Vero cells treated with trypsin or thermolysin (10 µg/mL). Notably, EjCoV-3 replicated to a considerable extent in Vero+transTMPRSS2 cells with no or low concentrations (1 µg/mL) of trypsin or thermolysin, although the virus titers in their presence were substantially higher than those in their absence. These data suggested that EjCoV-3 requires both hTMPRSS2 and exogenous proteases for efficient replication in cell culture.

### EjCoV-3 replicates in various human cell lines

To assess the potential of EjCoV-3 for human infection, we evaluated its growth in various human cell lines. Because EjCoV-3 was detected in bat feces rendering a possible growth in intestinal cells, we inoculated human rectal adenocarcinoma HRT18G cells or human colon adenocarcinoma CaCo-2 cells with EjCoV-3 and incubated them with trypsin (0, 1, or 10 µg/mL) or thermolysin (0, 1, or 5 ug/mL) (Fig. 6C). EjCoV-3 efficiently replicated in these cells at high concentrations. Because the respiratory route is likely responsible for the bat-to-human transmission of bat viruses, we next examined EjCoV-3 replication in RPMI 2650 cells, a human nasal squamous carcinoma cell line derived from the respiratory tract. EjCoV-3 showed efficient replication in RPMI2650 cells with high concentrations of trypsin or thermolysin. These data demonstrate that EjCoV-3 could replicate in human respiratory and intestinal cells, suggesting its potential to infect humans.

### EjCoV-3 replication in animal models

**Fig 7:**
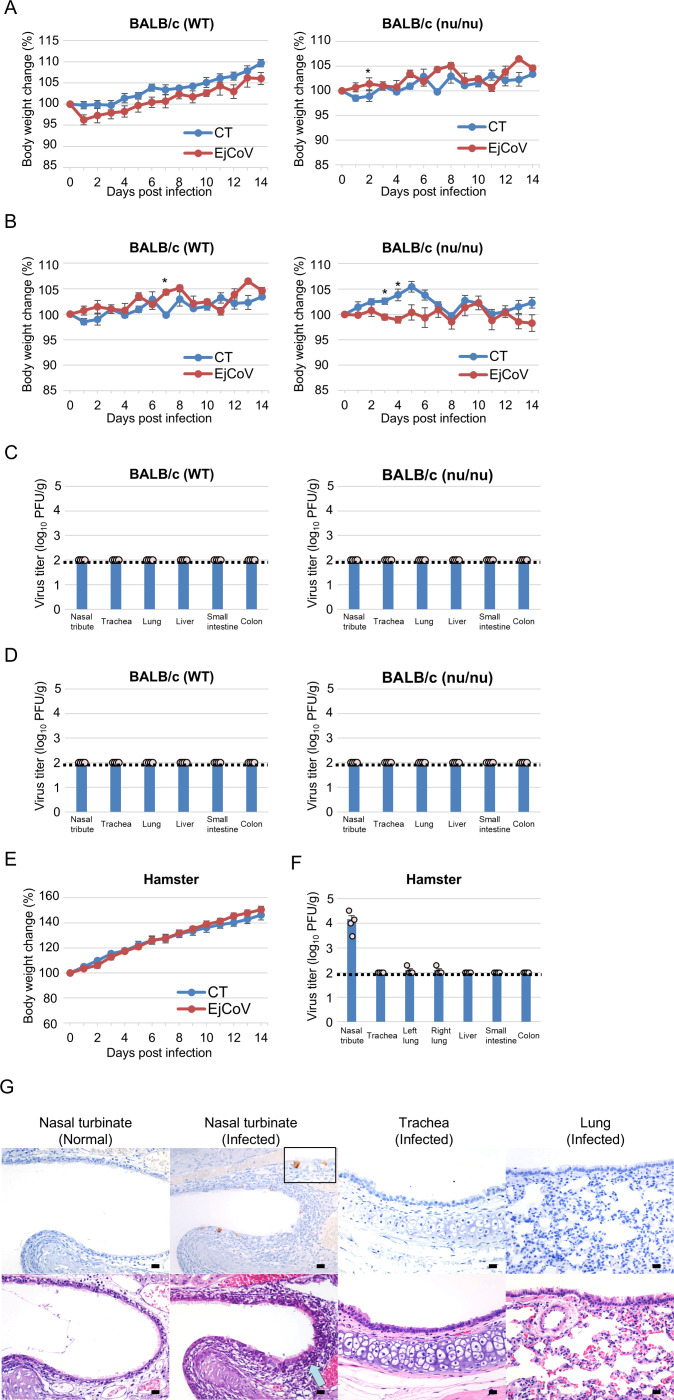
*In vivo* characterization of EjCoV-3 in mice and hamsters. Six-week-old female BALB/c and BALB/c nu/nu mice were inoculated with EjCoV-3 intranasally (2.7 × 10^4^ PFU) (**A**) or orally (5.4 × 10^4^ PFU) (**B**), respectively, and the body weight changes were monitored for 14 days. The results are reported as the mean values with standard errors. The asterisks indicate significant differences (*P < 0.05 by Student’s *t*-test). Viral replication in the mice intranasally (**C**) or orally (**D**) infected with EjCoV-3 was examined. At 3 days post-infection, four mice were euthanized, and the virus titers in each tissue were determined. The results are reported as the mean titers with standard errors, with a scatter plot of four values. A dotted line indicates the detection limit. (**E**) Four-week-old female Syrian hamsters were inoculated with EjCoV-3 both intranasally (5.4 × 10^4^ PFU) and orally (1.1 × 10^5^ PFU), and the body weight changes were monitored. Body weights were normalized to day 0. The results are reported as the mean values with standard errors. (**F**) Viral replication in the hamsters infected with EjCoV-3 was examined. At 3 days post-infection, four hamsters were euthanized, and the virus titers in each tissue were determined. The results are reported as the mean titers with standard errors, with a scatter plot of four values. A dotted line indicates the detection limit. (**G**) To detect virus antigens in tissues of the EjCoV-3-infected hamsters by immunohistochemistry, the nasal turbinates, trachea, and lungs were collected on day 3 post-infection. Viral antigens were stained using mouse anti-N antibody (upper column; brown staining). The pale blue arrow indicates the inflammatory infiltrates. A larger scaled image was also shown in a square box for the nasal turbinate. Hematoxylin and eosin stains for each equivalent tissue area were shown in the lower column. Uninfected hamster tissues served as negative controls (normal). Scale bars, 50 µm.

To assess the *in vivo* replication competence of EjCoV-3, we inoculated it either intranasally or orally into wild-type BALB/c mice, BALB/c nude mice, or intranasally and orally in Syrian hamsters, followed by body weight monitoring for 14 days. Furthermore, we collected respiratory and intestinal tissues 3 days post-inoculation and measured viral titers. Weight loss was not observed in either wild-type or nude mice, and the viral titer was negative in all of the tissue samples (Fig. 7A through D). In hamsters, although no weight loss was observed (Fig. 7E), the viruses were recovered in the nasal turbinate but not in other respiratory or intestinal organs (Fig. 7F). Viral antigens were detected in ciliated epithelial cells of the nasal turbinate through immunohistochemistry (IHC) (Fig. 7G). Inflammatory responses with infiltration of lymphocytes and plasma cells were also observed near the virus antigen-positive cells. Viral titers and antigen were negative in the trachea and lungs. These data suggested that the Syrian hamster is susceptible to EjCoV-3, indicating its potential to infect mammals via the respiratory route.

## DISCUSSION

This study characterizes the bat merbecovirus EjCoV-3 strain detected in Japan to evaluate its spillover potential. Although cultivable infectious EjCoV-3 could be a strong tool for such analyses, the virus could not be isolated from cell culture, posing a challenge to the generation of infectious EjCoV-3 through reverse genetics. To establish our system, we first analyzed the cell entry mechanisms of the virus using a pseudotyped virus system with the EjCoV-3 S protein and found that activation of the S protein by proteases was crucial for efficient cell entry of EjCoV-3. Cellular hTMPRSS2 and treatments with exogenous proteases, such as trypsin and thermolysin, could enhance its entry into cell culture. Based on these findings, we established a BAC-based reverse genetics system for EjCoV-3 using a specified culture method supplemented with proteases, leading to the successful generation of recombinant wild-type EjCoV-3. Using this rescued virus, we analyzed receptor molecules for infection and found that EjCoV-3 did not use DPP4 and ACE2, which are receptor molecules for MERS-CoV and SARS-CoV, respectively. The recombinant EjCoV-3 could replicate in human respiratory and intestinal cell cultures in the presence of trypsin or thermolysin, as well as in the upper respiratory tract of hamsters. These data suggest that EjCoV-3 can directly infect other mammals, including humans.

MERS-CoV and bat or pangolin merbecoviruses such as HKU4, NL140422, and HKU25 use human DPP4 (hDPP4) as a receptor (13, 15, 17–19). In contrast, African bat-derived PDF-2180 and NeoCoV reportedly use bat ACE2 as receptors (21). In addition, recent studies have shown that other merbecoviruses, such as HKU5 and MOW-15, are also capable of engaging ACE2 (22–24). As we previously reported, EjCoV-3 retains only one DPP4-contacting residue identified in MERS-CoV (Fig. S3A). In contrast, it possesses 11 ACE2-contacting residues found in HKU5-19s, suggesting a potential for ACE2-mediated cell entry (Fig. S3A). A recent preprint further reported that the EjCoV-3 spike weakly binds to human ACE2 and mediates entry in pseudovirus assays (39). While these findings suggest that EjCoV-3 may utilize ACE2 under certain conditions, our study clearly demonstrates that spike-mediated infection by EjCoV-3 occurs independently of both ACE2 and DPP4. This indicates that EjCoV-3 employs an alternative, currently unidentified receptor for cell entry. These results not only refine our understanding of receptor usage in EjCoV-3 but also point to the existence of a novel entry mechanism that could extend to other merbecoviruses.

Although the bat merbecovirus HKU4 SM3A strain and MERS-CoV chimeric virus (MERS-Uganda) with swapped extracellular regions of PDF-2180 S protein did not replicate in human respiratory cells (20, 30), EjCoV-3 replicated in human respiratory cells used in this study. In addition, EjCoV-3 replicated efficiently in the upper respiratory tract of hamsters. The molecular basis of these unique characteristics of EjCoV-3 remains unknown. As a binding molecule other than DPP4, sialylglycans on the cell surface have been reported to enhance the growth of MERS-CoV in respiratory cells (40). Interestingly, the amino acid residues of the S protein that bind to sialic acid were completely conserved in the S proteins of MERS-CoV and EjCoV-3 (Table S1), unlike those of HKU4 and HKU5, suggesting that the S protein of EjCoV-3 likely binds to sialic acid. The distribution of sialylglycan types on respiratory cells may explain the different susceptibilities of EjCoV-3 between mice and hamsters (41).

The S proteins of several coronaviruses are cleaved at the S1/S2 site intracellularly by ubiquitous furin or extracellularly by secreted proteases. Binding of the cleaved S protein to receptors induces a conformational change in the S protein, increasing the accessibility of TMPRSS2 and other proteases to cleave at the S2' site (28, 35–37). Previous reports showed that bat merbecoviruses, as well as bat sarbecoviruses, required cleaved S protein by high concentrations of extracellular trypsin prior to infection (13, 17, 18, 30, 42). Here, we found that EjCoV-3 required a high concentration of exogenous proteases (trypsin or thermolysin) in addition to intracellular TMPRSS2 for efficient growth in cell culture. The infectivity of EjCoV-3 also increased with trypsin treatment after viral attachment to the cell surface, postulating a conformational change in its S protein by binding to the receptor, followed by enhanced accessibility of the protease. The S proteins of MERS-CoV and various other coronaviruses have S1/S2 cleavage sites that consist of multiple basic amino acids and are cleaved by furin, whereas that of EjCoV-3 has only a single arginine residue at this site (Fig. S3B and C), suggesting that it was not intracellularly cleaved by furin. Treatment with exogenous thermolysin resulted in cleavage of the EjCoV-3 S protein in a concentration-dependent manner, and the infectivity of the pseudotyped virus with the EjCoV-3 S protein correlated with the degree of cleavage, suggesting that cleavage by thermolysin induced a conformational change in the S protein, which increased the accessibility of TMPRSS2 to the S2' site. In contrast, treatment with low concentrations of trypsin did not lead to enhanced viral infectivity, suggesting that the cleavage site was different from that produced through thermolysis. Although the S proteins were highly cleaved into smaller sizes after treatment with high concentrations of trypsin, infectivity markedly increased. Based on these observations, it is concluded that multiple cleavage sites of the S protein are responsible for the efficient growth of EjCoV-3. Although we identified the thermolysin-mediated cleavage site (Fig. 1D; Fig. S3D), the precise trypsin-mediated cleavage sites and the potential synergistic effects of combined protease cleavability remain to be elucidated.

Host specificity and tissue tropism are often determined by both receptor usage and the cleavability of viral glycoproteins. For example, SARS-CoV-2 can infect hamsters but not mice due to the absence of a compatible receptor (43–45). Similarly, MERS-CoV does not infect mice and hamsters, although transgenic mice expressing human DPP4 are susceptible (46–49). In the present study, EjCoV-3 grew efficiently in the nasal turbinate of hamsters but not in mice. This finding may be because hamsters possess compatible receptors and proteases responsible for EjCoV-3 replication. Regarding the protease-mediated S protein cleavage, it is possible that a hamster-derived protease expressed in the nasal ciliated epithelium cleaved the S protein, as reported in SARS-CoV-2 infection in humans (50). It is also possible that, similar to SARS-CoV (51), the S protein is cleaved by proteases derived from bacteria in the nasal turbinate, suggesting that EjCoV-3 could replicate in the upper respiratory tract of animals other than bats. Since EjCoV-3 did not grow in the intestinal tracts of hamsters and mice, it likely replicates in the intestinal tract of bats, as it has been previously detected in bat feces. Previous reports have shown that a high concentration of trypsin is present in the intestinal tract (52), and mouse hepatitis virus, a betacoronavirus, replicates in the intestinal epithelium using trypsin (53), suggesting that EjCoV-3 might grow in the intestinal tract of hosts other than bats, including humans.

In summary, we successfully generated recombinant cultivable EjCoV-3 using newly established reverse genetics with a specified culture method supplemented with proteases. Our findings revealed that EjCoV-3 replicated in human respiratory and gastrointestinal cell cultures in the presence of trypsin or thermolysin and in the nasal turbinate of hamsters, suggesting that this bat virus has the potential for transmission to other mammals, including humans, via the airborne route with certain adaptation. Aside from previous studies focusing on the evaluation of the potential of bat merbecoviruses to infect humans using hACE2 and hDPP4, this study provides evidence that bat merbecoviruses may replicate in the upper respiratory tract of humans in a hACE2- and hDPP4-independent manner. Therefore, it is important to elucidate the molecular mechanisms underlying the transmission capacity of bat merbecoviruses for future pandemic preparedness. The findings and technologies obtained in this study could contribute to the characterization of other merbecoviruses.

## MATERIALS AND METHODS

### Cells

Vero/TMPRSS2 cells (kindly gifted by Dr. Makoto Takeda, National Institute of Infectious Disease, Japan), Vero cells (RIKEN BRC, #RCB0001), Vero-ACE2KO cells (38), human embryonic kidney 293T (HEK293T) cells (RIKEN BRC, #RCB2202), and human rectal tumor 18G (HRT18G) cells (ATCC, #CRL-11663) were maintained in Dulbecco’s modified Eagle’s medium (DMEM; Nacalai Tesque, Kyoto, Japan) supplemented with 10% fetal bovine serum (FBS), 100 units/mL penicillin, and 100 µg/mL streptomycin. Human nasal squamous carcinoma RPMI2650 cells (JCRB Cell Bank, #JCRB9058) were maintained in minimal essential medium (MEM) supplemented with 10% FBS, 1% non-essential amino acids (NEAA), 50 units/mL penicillin, and 50 µg/mL streptomycin. Human colon adenocarcinoma CaCo-2 cells (RIKEN BRC, #RCB0988) were maintained in MEM supplemented with 20% FBS, 1% NEAA, 100 units/mL penicillin, and 100 µg/mL streptomycin.

### Plasmids

We deleted a part of the S gene sequence encoding a 16-aa at the C-terminus of the S protein of MERS-CoV (HCoV-EMC/2012, GenBank accession no. NC_019843) (54) and EjCoV-3 (GenBank accession no. LC706865) (16) and fused with a glycine-serine linker (GSSG) and a FLAG-tag at the C-terminus in each cloning plasmid. These were subcloned into the pCAGGS vector (pCAGGS-EjCoV-3-S-del16-FLAG and pCAGGS-MERS-CoV-S-del16-FLAG, respectively). Similarly, the S protein gene of SARS-CoV with a 19-aa deletion at the C-terminus (55) was cloned into the pCAGGS vector (pCAGGS-SARS-S-St19). The vesicular stomatitis Indiana virus glycoprotein (G) expression plasmid (pCAGGS-VSV-G) was also used. The cDNA of human (h) TMPRSS2, hACE2, or African green monkey (agm) DPP4 was cloned into a pS lentiviral vector (pS-hTMPRSS2, pS-hACE2, or pS-agmDPP4, respectively) (56).

### Generation of Vero-DPP4KO cells

Vero-DPP4KO cells were generated using the CRISPR-Cas9 system. The target sequence was cloned into plentiCRISPR plasmids (Addgene plasmid #52961, a gift from Dr. Feng Zhang) (57) using a DNA Ligation Kit (Takara Bio, Shiga, Japan). Vero/TMPRSS2 cells were transfected with DPP4 gene-targeting plasmid using polyethylenimine (PEI; Polysciences, Warrington, PA, USA). At 24 h post-transfection, 10 µg/mL puromycin was added to the medium to select KO cells. The cell clones were isolated using a limiting dilution method, and those with in/dels in the target genes were selected. Loss of DPP4 expression in Vero-DPP4KO cells was confirmed through western blotting using an anti-human DPP4 antibody (Abcam, #ab28340, Cambridge, England).

### Addback or transduction of protease genes by the lentivirus vector system

To generate recombinant lentiviruses, we co-transfected pS-hTMPRSS2, pS-hACE2, or pS-agmDPP4 with p8.9QV (56) and pCAGGS-VSV-G plasmids into HEK293T cells. Vero cells infected with hTMPRSS2-expressing lentiviral vectors were named as Vero+transTMPRSS2. Vero-ACE2KO or Vero-DPP4KO cells infected with the hACE2 or agmDPP4-expressing lentiviral vectors were named ACE2- or DPP4-addback cells. The expression of hTMPRSS2 in Vero+transTMPRSS2 cells, agmDPP4 in DPP4-addback cells, and hACE2 in ACE2-addback cells was confirmed by western blotting using anti-human DPP4, anti-human TMPRSS2 (Sigma-Aldrich, #HPA035787, MO, USA), and rabbit anti-ACE2 (Abcam, #ab15348, Cambridge, England) antibodies, respectively.

### Production of VSV pseudotyped virus with S proteins

VSV pseudotyped viruses, with attached coronavirus S proteins, were produced in HEK293T cells using VSVΔG*-GFP, which possesses a GFP reporter gene instead of the viral G gene, as described previously (55, 58, 59). HEK293T cells were transfected with pCAGGS-EjCoV-3-S-del16-FLAG, pCAGGS-MERS-CoV-S-del16-FLAG, pCAGGS-SARS-CoV-S-St19, pCAGGS-empty, or pCAGGS-VSV-G. At 24 h post-transfection, cells were incubated with VSVΔG*-GFP for 1 h, washed twice, and incubated with Opti-MEM for 24 h. The supernatant was collected, centrifuged, filtered through a 0.45 µm filter to remove cells and cell debris, and stored at −80°C until use. The VSV pseudotyped viruses with the S proteins of EjCoV-3, MERS-CoV, SARS-CoV, VSV-G, and empty controls were designated as VSV/EjCoV-3, VSV/MERS-CoV, VSV/SARS-CoV, VSV/VSV-G, and VSV/empty, respectively. VSV/EjCoV-3 was incubated with various concentrations of TPCK-treated trypsin (Worthington, Lakewood, NJ, USA), dissolved in PBS, for 1 h at 21°C. After incubation, 500 µg/mL soybean trypsin inhibitor (Nacalai Tesque) in PBS was added to stop the reaction. VSV/EjCoV-3 was incubated with various concentrations of thermolysin (Nacalai Tesque, Kyoto, Japan) dissolved in DMEM in the presence of 50 mM CaCl_2_ for 1 h at 21°C. VSV/EjCoV-3 treated with or without proteases, VSV/MERS-CoV, VSV/SARS-CoV, and VSV/empty were incubated with the anti-VSV-G neutralizing antibody I1 (60) for 30 min at 21°C to neutralize the remaining VSVΔG*-GFP.

### Purification of protease-treated VSV/EjCoV-3

VSV/EjCoV-3 was incubated with trypsin or thermolysin, as described above, and then a soybean trypsin inhibitor or 100 mM EDTA was added to stop the reactions. The protease-treated VSV/EjCoV-3 was layered onto a 20%, 30%, 50%, and 60% discontinuous sucrose gradient and ultracentrifuged at 110,000 × *g* for 3 h at 4°C using a P32ST rotor (Eppendorf Himac Technologies, Ibaraki, Japan). The virus-containing interface between the 30% and 50% sucrose was collected, diluted in PBS, and ultracentrifuged at 110,000 × *g* for 3 h at 4°C using a P32ST rotor. Purified virus pellets were suspended in 30 µL of 2× Laemmli SDS sample buffer and subjected to western blot analysis. FLAG-tagged S proteins were detected using a mouse anti-FLAG monoclonal antibody (Medical & Biological Laboratories, Tokyo, Japan). For N-terminal protein sequencing, purified VSV/EjCoV-3 was lysed, and the S protein was isolated using the DYKDDDDK Fab-Trap Agarose Kit (ChromoTek, Martinsried, Germany). The purified S protein was deglycosylated with PNGaseF, subjected to SDS-PAGE, and transferred to a PVDF membrane. The membrane was stained with crystal violet, and the corresponding protein band was excised. N-terminal amino acid sequencing was performed using a PPSQ-31A protein sequencer (Shimadzu, Kyoto, Japan).

### Cell entry assay

The cells seeded on 12-well plates were inoculated with various VSV pseudotyped viruses for 1 h at 37°C. After incubation, the inoculum was removed, and the cells were washed once with DMEM supplemented with 1% FBS (DMEM/1% FBS) and maintained with DMEM/1% FBS for 20 h at 37°C. To test the cathepsin requirement for viral cell entry, Vero or Vero+transTMPRSS2 cells were incubated with 25 µM of cathepsin B/L inhibitor E-64d (Tokyo Chemical Industry, Tokyo, Japan) for 2 h at 37°C and then inoculated with either VSV/VSV-G, VSV/MERS-CoV, trypsin-treated (500 µg/mL) VSV/EjCoV-3, or thermolysin-treated (100 µg/mL) VSV/EjCoV-3. After incubation for 1 h at 37°C, the cells were maintained in DMEM/1% FBS for 20 h at 37°C. To analyze the point of action of the protease, Vero cells were placed on ice for 15 min to stop endocytosis and then inoculated with trypsin-treated (500 µg/mL) or untreated VSV/EjCoV-3. After incubation for 1 h on ice, the cells were washed once and incubated in Opti-MEM with or without trypsin (25 µg/mL) for 30 min at 37°C. After incubation, the cells were maintained in DMEM/1% FBS for 20 h at 37°C. The number of GFP-positive cells within one microscopic field (3.1 mm^2^) was counted using an AxioVert.A1 fluorescent microscope (Carl Zeiss, Oberkochen, Germany). Virus titers were determined as the number of GFP-positive cells per well in a 12-well plate (3.9 cm^2^) by calculating from those for five microscopic fields (58). The virus titers are expressed as mean values with standard errors from three independent experiments.

### Reverse genetics system for EjCoV-3

To clone the cDNA of the full-length EjCoV-3 genome into a BAC (Fig. S2), the viral RNA was reverse-transcribed with random hexamer primers and amplified by PCR for 10 cDNA fragments. Each fragment was TA-cloned into the pTA2 vector using a TArget Clone kit (Toyobo, Osaka, Japan) and referred to as pTA2-EjCoV-3-1 through -10. The cytomegalovirus immediate early promoter was cloned into pSMART BAC (Lucigen, Wisconsin, USA), designated as pBAC-CMVp, and transformed into *Escherichia coli* SW102 strain (61). Ampicillin resistance gene cassette (AmpR) was amplified with 50 bp homology arms, and the cDNA of EjCoV-3 was amplified from pTA2-EjCoV-3-1 and -2 by PCR. The PCR products were then connected by PCR. SW102 cells containing pBAC-CMVp were heat-shocked to induce red recombinase expression, followed by electroporation with 100 ng of the PCR product. The recovered cells were plated on LB medium plates containing 12.5 µg/mL chloramphenicol and 50 µg/mL ampicillin at 32°C. The resulting BAC vector was named pBAC-CMVp-EjCoV-3-1-2-AmpR. The kanamycin resistance gene cassette (KnR) and cDNA of EjCoV-3 were amplified from pTA2-EjCoV-3-3, -4, and -5 by PCR, and the PCR products were connected by another PCR. SW102 cells containing pBAC-CMVp-EjCoV-3-1-2-AmpR were heat shocked to induce red recombinase, followed by electroporation with 100 ng of the PCR product. The recovered cells were plated on LB plates containing 12.5 µg/mL chloramphenicol and 25 µg/mL kanamycin. The resulting BAC vector was named BAC-CMVp-EjCoV-3-1-2-3-4-5-KnR. The residual cDNA of EjCoV-3, 25-nucleotide of poly A, hepatitis delta virus ribozyme, and bovine growth hormone polyadenylation signal were cloned into BAC in the same manner as described above using ampicillin and kanamycin. The resulting BAC vector was named pBAC-EjCoV-3. HEK293T cells seeded in 6-well plates were transfected with 2 µg pBAC-EjCoV-3 using 4 µL TransIt 293 (Mirus Bio, Madison, WI, USA). At 16 h post-transfection, the cells were washed twice and incubated with 1 mL Opti-MEM for 36 h. The supernatant containing the rescued virus was collected, centrifuged, and designated EjCoV-3 P0. EjCoV-3 P0 was incubated with 100 µg/mL thermolysin in the presence of 50 mM CaCl_2_ for 1 h at 21°C. Vero/TMPRSS2 cells were infected with the thermolysin-treated EjCoV-3 P0. After 1 h of incubation, the cells were washed once and maintained with DMEM/1% FBS for 12 h. Then, the cells were washed twice with MEM supplemented with 0.3% bovine serum albumin (MEM/BSA) and incubated with MEM/BSA supplemented with 10 µg/mL TPCK-treated trypsin for 24 h. The supernatant was collected, centrifuged, and labeled EjCoV-3 P1. After passing it on Vero/TMPRSS2 using the above method, the P4 virus was collected, aliquoted, and stored at −80°C as the stock virus.

### Growth kinetics of EjCoV-3

Vero/TMPRSS2, Vero, or Vero+transTMPRSS2 cells were infected with EjCoV-3 at a multiplicity of infection (MOI) of 0.01. After incubation at 37°C for 1 h, the inoculum was completely removed, and the cells were maintained in DMEM/1% FBS. After incubation at 37°C for 12 h, the cells were washed twice using MEM/BSA and maintained in MEM/BSA with or without TPCK-treated trypsin (1, 2.5, 5, or 10 µg/mL for Vero/TMPRSS2 cells and 1 or 10 µg/mL for Vero and Vero+transTMPRSS2 cells, respectively), or thermolysin (1, 2.5, 5, or 10 µg/mL for Vero/TMPRSS2 cells and 1 or 10 µg/mL for Vero and Vero+transTMPRSS2 cells, respectively). Culture supernatants were collected every 12 h post-infection. The human cell lines were infected with EjCoV-3 at an MOI of 0.1. After incubation at 37°C for 1 h, the inoculum was removed, and the cells were maintained in DMEM/1% FBS. After incubation at 37°C for 12 h, the cells were washed twice with MEM/BSA and maintained in MEM/BSA with or without TPCK-treated trypsin (1 or 10 µg/mL) or thermolysin (1 or 5 µg/mL). Culture supernatants were collected daily for up to 4 days post-infection. To measure viral titers, plaque assays were performed in 12-well plates using Vero/TMPRSS2 cells. After virus adsorption to the cells at 37°C for 1 h, the inoculum was removed, and the cells were overlaid with MEM supplemented with 1% FBS, 2.5 µg/mL thermolysin, and 0.8% agarose. At 60–72 h post-infection, the agarose was removed, and the cells were fixed with methanol and stained with 0.1% crystal violet to count plaque numbers.

### Animal experiments

Six-week-old female BALB/c mice and BALB/c nu/nu mice were purchased from Japan SLC (Hamamatsu, Japan). After 1 week of adaptation, the mice were intranasally inoculated with 2.7 × 10^4^ PFU of EjCoV-3 (*n* = 8–9/group) or orally inoculated with 5.4 × 10^4^ PFU of EjCoV-3 (*n* = 8/group). The viral inoculum was derived from a stock virus propagated in Vero/TMPRSS2 cells with trypsin supplementation. The body weight and mortality of the mice were monitored for 14 days. Three days post-inoculation, four mice per group were euthanized, and the lungs, trachea, nasal turbinates, liver, small intestine, and colon were harvested and homogenized in MEM/BSA using a TissueLyser II (Qiagen). The homogenates were centrifuged, and supernatants containing the viruses were collected. Viral titers were determined using the plaque assay. Four-week-old female Syrian hamsters were purchased from Japan SLC. After 1 week of adaptation, they were inoculated with EjCoV-3 via both intranasal (5.4 × 10^4^ PFU) and oral (1.1 × 10^5^ PFU) routes (*n* = 8). The body weight and mortality of the hamsters were monitored for 14 days. Three days post-inoculation, four hamsters were euthanized, and the viral titers in each tissue were determined as described in the mice experiments.

### Histology and immunohistochemistry

The tissues were fixed in 10% neutral-buffered formalin and embedded in paraffin. Sections (2 µm in thickness) were stained with hematoxylin and eosin. For IHC, deparaffinized sections were incubated in methanol with 1% hydrogen peroxide for 15 min to block endogenous peroxidase activity. Antigen retrieval was performed by heating the sections in citrate buffer (pH 6.0) at 121°C for 10 min. The sections were blocked with 8% skim milk in TBS and then incubated with mouse anti-N antibody (clone 5F3) at 4°C overnight. Immunolabeled antigens were visualized using the EnVision+ system (Agilent, Santa Clara, CA) and reacted with 0.05% 3′3-diaminobenzidine and 0.03% hydrogen peroxide in tris-hydrochloric acid buffer. The sections were then counterstained with hematoxylin.

### Statistical analysis

Data sets on the viral growth kinetics (Fig. 6) and cell entry assay with the pseudotyped viruses (Fig. 2B, 4A, and B) were analyzed using the Holm method with two-tailed analysis to determine the statistical significance of the differences. Data sets for the cell entry assay with the pseudotyped viruses (Fig. 1A, B, and 3) were analyzed using Dunnett’s test to determine the statistical significance of the differences. Data sets on the body weights of mice (Fig. 7A and B) and hamsters (Fig. 7E) were analyzed using Student’s *t*-test with a two-tailed analysis.

## Data Availability

No data sets were deposited in public repositories. All data generated or analyzed during this study are included in this published article. Further information is available from the corresponding author upon reasonable request.

## SUPPLEMENTAL MATERIAL

Supplemental material jvi.00727-25-s0001.pdf Figures S1 to S3; Table S1.

ASM does not own the copyrights to Supplemental Material that may be linked to, or accessed through, an article. The authors have granted ASM a non-exclusive, world-wide license to publish the Supplemental Material files. Please contact the corresponding author directly for reuse.
