# Supplementary material for: In vitro and in vivo characterization of a bat merbecovirus with ACE2- and DPP4-independent cell entry
Source: J Virol. 2025 Jun 17;99(7):e00727-25. doi: 10.1128/jvi.00727-25 (PMC12282129; doi:10.1128/jvi.00727-25)
Supplement: Supplemental material — Figures S1 to S3; Table S1. [file jvi.00727-25-s0001.pdf]

Supplementary Information

In vitro and in vivo characterization of a bat MERS-related coronavirus with DPP4-independent cell entry

Hiromichi Matsugo, Tomoya Kitamura, James Chambers, Naohiro Takahashi, Ayano Ichikawa, Misa Katayama, Kaixin Li, Wataru Sekine, Kosuke Ohira, Hiroho Ishida, Akiko Takenaka-Uema, Kazuyuki Uchida, Masayuki Shimojima, Taisuke Horimoto, Shin Murakami

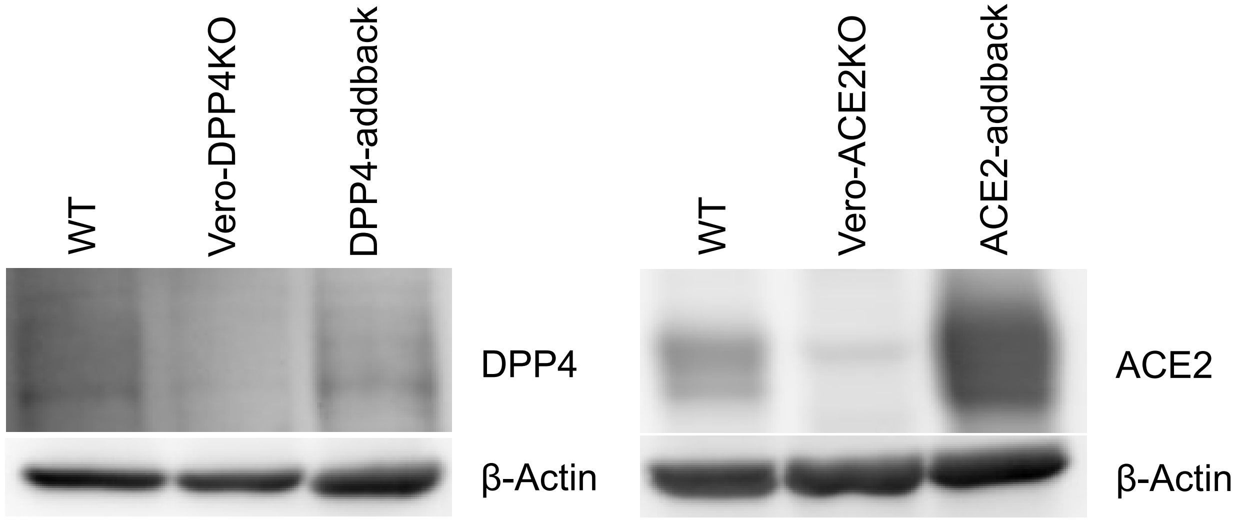

Fig. S1. Western blot analysis of DPP4 or ACE2 knockout cells. The knockout of DPP4 in Vero-DPP4KO cells or ACE2 in Vero-ACE2KO cells and the expression of DPP4 in DPP4-addback cells or ACE in ACE2-addback cells were confirmed by western blotting analysis.

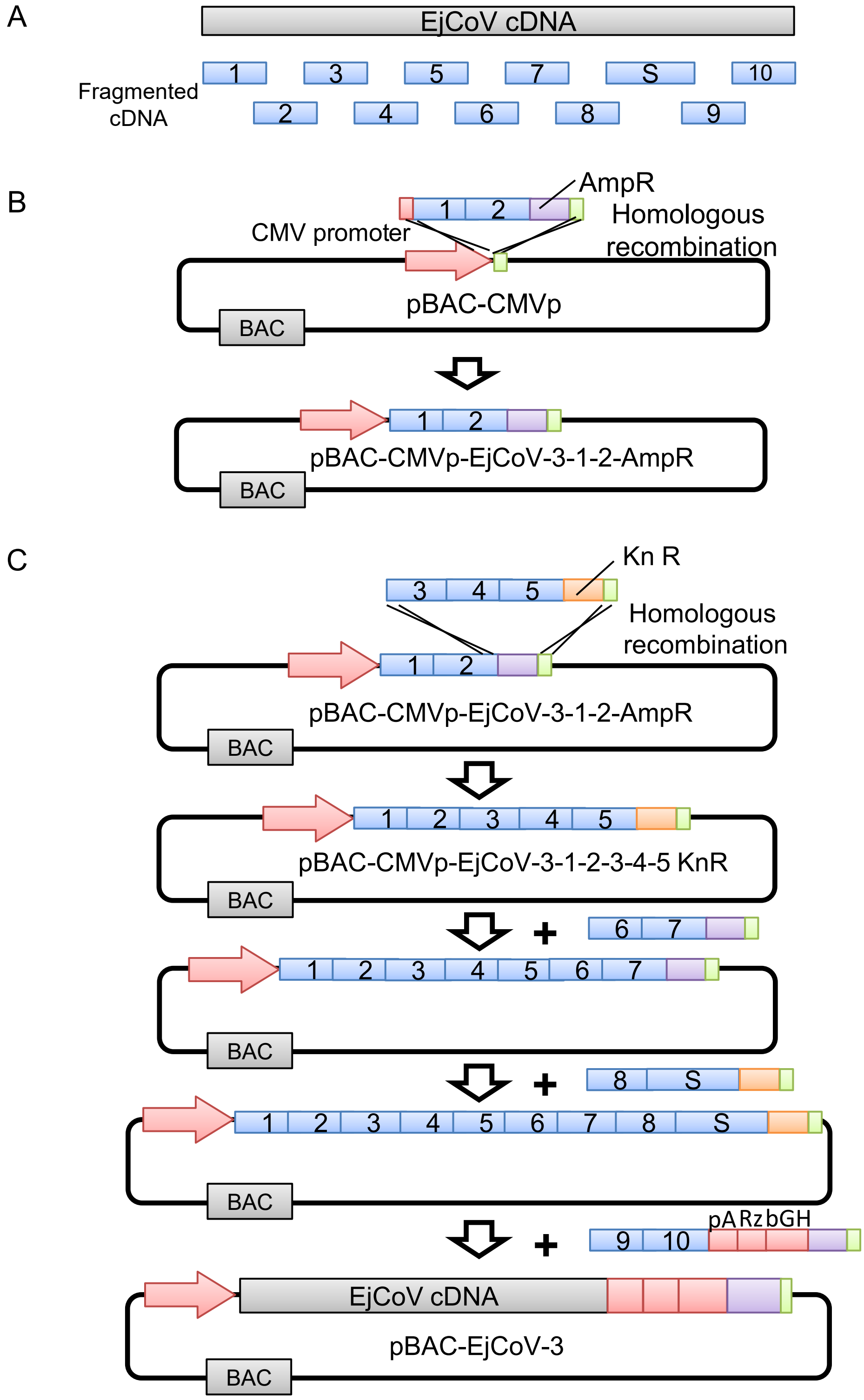

Fig. S2. Cloning of the EjCoV cDNA into BAC vectors. (A) Schematic representation of EjCoV cDNA fragment. (B) The cytomegalovirus immediate early promoter (CMVp) was cloned into pSMART BAC and named pBAC-CMVp,The cDNA fragment 1, 2 and ampicillin resistance gene cassette (AmpR) were inserted into pBAC-CMVp by homologous recombination. The resultant BAC was named pBAC-CMVp-EjCoV-3-1-2-AmpR. (C) The residual cDNA of EjCoV, 25-nucleootide of polyA (pA), hepatitis delta virus ribozyme (Rz), and bovine growth hormone polyadenylation signal (bGH) were cloned into BAC by homologous recombination using ampicillin and kanamycin in turn. The resultant BAC vector was named pBAC-EjCoV-3.

A

```
DPP4        MERS-CoV        484:  VPHNLTTITKPLKYSYINKCS------RLLSDDRT-EVPQLVNANQ--YSP---CVSIVPST-VWEDGDYYRK-----QLSPLEGGGWLVASGSTV------AM-TEQL
-binding    HKU4-SM3A       489:  VPANVT-ITKPDAYGYISKCS------RLTGANQDVETPLYINPGE--YSI---CRDFSPGG-FSEDGQVFKR-----TLTQFEGGGLLIGVGTRV------PM-TDNL
            NL140422        488:  APANLT-ITKPSSYSFISKCS------RLTGDNSHIETPIVINPGE--YSI---CKNFAPNG-FSQDGDYFTR-----QLSQLEGGGILVGVGSVT------PM-TDTL
            HKU25-YD131305  491:  APANIT-LTKPNNYNWLTECY------HSTAFGKQ---PYYVQPGQ--YTP---CLGLAISG-FTTSYE--TR----RDPNTKMAATGLVT------AM-TTSL
            EjCoV-3         490:  VPSNIS-LTKPTNYNWLTECY------KSSAFGKQ---PFYVQPGQ--YTP---CLGLAISG-FSNSYE--SR----QDPNTKTTATGYVA------PM-TNNL
ACE2        HKU5-19s        493:  VPQNLTTITKPSNYAYLTECY------KTSAYGKN---YLYNAPGG--YTP---CLSLASRG-FSTKYQ--SH-------SD----GELTTTGYIY------PV-TGNL
-binding    NeoCoV          487:  VNSSIG-ISYAGAYSYITNCN------YGATNKDD-----VVKPGGRASQQ---CITGALNSPTTGQLWA-----YNFGGVPYRVSRLTYT------DHLSDPL
            MOW15-22        487:  VPLGGVN---PTNYTTLTNCYGCVDKDPANPWGDQICIPEFVTEVEPGFRPKPSCARVGLEG-HISGNDTYSAIVTNGELDSTGDPIWRKGVALTKQPIDSSRA
```

B

```
MERS-CoV        737:  ALPDTPSTLTPRSVR---SVPGEMRLASIAFNHP
EjCoV-3         735:  AVPSTA--------R---SSASPLQLATINYTQP
SC2013          708:  AVPSPN-------IRVVSGDPQPMQLATINFTQP
HKU25-YD131305  736:  AVPFVT--------R---DTSGSLQLAVINFTRP
HKU25-NL140462  737:  AVPSKN--------R---DTTSNLQLAVINFTRP
GD2013          735:  AIPPTTSS---RLRRATSGVPDVFQIATLNFTSP
HKU4-SM3A       741:  AVPPVS------TFR--SYSASQFQLAVLNYTSP
HKU5-19s        735:  AIPPNPSA---RLARASSGVTDVFQIATLNFTSP
NeoCoV          734:  AIPPNTNL---RSGR------STFGLGSLAYNSP
PDF-2180        735:  AIPPNTNL---RVGR------STFGLGSLAYNSP
NL13845         743:  AVPATT------KGR--SASSGYLQLATINYTQP
NL140422        742:  AVPSTT--------R----SSSQLQLATINYTQP
MOW15-22        753:  AIPPSP------HSR---TNSG-FALSLIAYPEP
206645-40       733:  AVPPTS-----STAR---TTSSEFQLASINFNQP
```

C

```
MERS-CoV        870:  NLTLLEPVSISTGSRSARSAIEDLLFDKVTIADP
EjCoV-3         860:  NLTLLQVPKVVTSEYSKRSAIEDLLFNKVTIADP
SC2013          837:  NLTLLQVPKVVTSEYSYRSAIEDLLFNKVTIADP
HKU25-YD131305  861:  NLTLLQVPKVTTSQYSYRSTIEDLLFNKVTIADP
HKU25-NL140462  862:  NLTLLQVPKVTTSQYSYRSTIEDLLFNKVTIADP
GD2013          868:  NLTMLQIPQVTTGERKYRSAIEDLLFNKVTIADP
HKU4-SM3A       869:  NLTLLQVPQIGGSSSSYRSAIEDLLFDKVTIADP
HKU5-19s        868:  NLTMLQIPQVTTGERKYRSAIEDLLFNKVTIADP
NeoCoV          861:  NLTLLEPLSVSTGSSNARSALEELLFDSVTIADP
PDF-2180        862:  NLTLLEPLSVSTGSSNARSALEELLFDKVTIADP
NL13845         871:  NLTMLQVPKVVTSEYSKRSAIEDLLFNKVTIADP
NL140422        866:  NLTLLQVPQVSTSQYSHRSAIEDLLFNKVTIADP
MOW15-22        879:  NLSLLEPLNVQTSQYGYRSAIEDLLFNKITIADP
206645-40       861:  NLTLLQVPEVTTSQYSYRSAIEDLLFNKITIADP
```

D

```
EjCoV-3         681:  TMMHQFSRTTQATLRMRVSSNTGPLQTTVGCALG
MERS-CoV        684:  STMSQYSRSTRSMLKRR-DSTYGPLQTPVGCVLG
SC2013          654:  TMMQQFSRMTQSRLRMRISDDSGPLQTAVGCAIG
HKU25-YD131305  682:  TMSTQFSRMTQANLRAR--AANQPIQTAVGCAIG
HKU25-NL140462  683:  TMTTQLSRMTQAKLRSR--SFTTPIQTAVGCVIG
GD2013          683:  TMMSQFSRMTKTNLLAR--TTPGPLQTTVGCAMG
HKU4-SM3A       689:  TMMSQFSRLTQSNLRRR--DSNIPLQTAVGCVIG
HKU5-19s        683:  TMMSQFSRMTKTNLLAR--TTPGPLQTVVGCAMG
NeoCoV          682:  TMAAQFSRETRASLVSR--NMQNLLQTSVGCVMG
PDF-2180        683:  TMAAQFSRETRASLVSR--NMQNLLQTSVGCVMG
NL13845         689:  TMMSQFSRDTQVLLRRRDASSAGPLQTTVGCVLG
NL140422        688:  TMLHQFSRTTQASLRMRDVSNSGPLQTAVGCVIG
MOW15-22        702:  SFMSQFSRETQSLLRRR---DVGPLQSPVGCLIG
206645-40       682:  TFMPQFSRMTQSALRMR---STGPIQTPVGCAMG
```

Fig. S3 Alignment of amino acid sequences of the S proteins from merbecoviruses. Putative receptor-binding motif (A) of the S proteins of MERS-CoV (GenBank accession no. NC_019843), bat merbecovirus EjCoV (LC706865), HKU4-SM3A (MW218395), NL140422 (MG021452), HKU25-YD131305 (KX442564), HKU5-19s (AGP04932), NeoCoV (KC869678), and MOW15-22 (ON325306) are shown. Spikes with DPP4-binding ability are highlighted in orange, whereas those with ACE2-binding ability are in blue. DPP4-contacting residues in MERS-CoV are indicated in blue, while ACE2-contacting residues in HKU5-19s, NeoCoV, and MOW15-22 are in red. Alignment of the putative cleavage site of S1/S2 (B), S2' (C), and thermolysin (D) of the S proteins of MERS-CoV, bat merbecovirus EjCoV-3, SC2013 (KJ473821), HKU25-YD131305, HKU25-NL140462, GD2013 (KJ473820), HKU4-SM3A, HKU5-19s, NeoCoV, PDF-2180 (KX574227), NL13845 (MG021451), NL140422, MOW15-22, and 206645-40 (MG596802) are shown. Arrows indicate the cleavage site.

Supplementary Table. Alignment of contacting amino acid residues to sialic acid identified in MERS-CoV.

	36	39	91	92	101	131	132	133	307
MERS-CoV	Q	F	H	A	F	I	I	S	R
EjCoV-3	Q	F	H	A	F	I	I	S	R
SC2013	R*	F	H	A	F	I	I	S	R
HKU25-YD131305	Q	Y	H	A	F	I	I	S	R
HKU25-NL140462	Q	Y	H	A	F	I	I	S	R
GD2013	R	Y	H	S	F	V	I	S	R
HKU4-SM3A	A	F	H	A	Y	V	I	S	R
HKU5-19s	Q	F	H	S	F	I	I	S	R
NeoCoV	Q	Y	H	K	F	I	V	G	R
PDF-2180	Q	Y	E	K	F	I	V	G	R
NL13845	N	Y	H	A	F	I	I	S	R
NL140422	S	F	H	S	F	I	I	S	R
MOW15-22	Q	Y	H	T	F	I	I	S	R
206645-40	D	A	H	A	Y	V	I	S	R

*Conserved amino acids are highlighted in red and unconserved amino acids are highlighted in green.
